# Safety and Efficacy of the VariLift-C® Cervical Standalone Interbody Fusion Device with Emphasis on Multiple-level and Prior Fusion Cases

**DOI:** 10.7759/cureus.5885

**Published:** 2019-10-10

**Authors:** Georgios A Maragkos, Rouzbeh Motiei-Langroudi, Jeffrey Arle

**Affiliations:** 1 Neurosurgery, Icahn School of Medicine at Mount Sinai, New York, USA; 2 Neurosurgery, University of Kentucky, Lexington, USA; 3 Neurosurgery, Beth Israel Deaconess Medical Center | Harvard Medical School, Boston, USA

**Keywords:** anterior cervical discectomy and fusion, acdf, standalone cage, expandable cage, cervical degenerative disc disease, varilift-c

## Abstract

Introduction

The VariLift-C^®^ is a stand-alone, expandable, cervical interbody fusion device, not requiring the addition of anterior plating. Because of the access and placement technique, as well as not needing a plate, the device could potentially be used preferentially for patients with prior anterior fusions or for multiple, non-contiguous vertebral segments.

Methods

A retrospective chart review was conducted and all cases of anterior cervical discectomy and fusion (ACDF) using VariLift-C^®^ implants by a single surgeon were included. Patient baseline and operative characteristics were collected, and their follow-up notes were searched for outcomes. Descriptive statistics are provided.

Results

Seventy-one patients were included in this study, 14 of which had had a prior fusion, and 32 underwent a multilevel ACDF. A total of 108 cervical levels were fused. Mean age (± SD) at surgery was 50.3 ± 11.4 years and mean (± SD) follow-up was 6.5 ± 10.7 months. There were 39 single-level, 27 two-level and five three-level fusions. Four cases (5.6%) underwent multilevel re-operations. Thirty-three patients (80.3%) reported substantial improvement in their symptoms on follow-up, 19 of whom (26.8%) had no residual symptoms. Only two patients (2.8%) reported a worsened condition after surgery. There were 10 cases (12.8%) of postoperative neurologic deficit, one case of dysphagia and three cases of vocal cord paresis.

Conclusions

These results display the use of VariLift-C^®^ for symptomatic cervical degenerative disorders, with a focus on fusion extension or multiple-segment ACDF procedures. Our experience with favorable self-reported outcomes and low complication rates showcases the safety and efficacy of the VariLift-C^®^ device for ACDF.

## Introduction

VariLift-C® is a stand-alone, expandable cervical interbody fusion device, that does not require the addition of anterior plating. There are many studies examining the efficacy and safety of standard anterior plating techniques for performing anterior cervical spine discectomy and fusion (ACDF) [1]. However, using a stand-alone interbody fusion device may require less tissue dissection and minimize technical risks, by removing the need to add anterior plating and screws on the vertebral bodies. Furthermore, when operating on a new level, in patients with a prior fused level, using a stand-alone device, such as the VariLift-C® device, obviates the need to remove any previously placed anterior plates, potentially reducing operative risk, tissue dissection and surgical time.

The objective of this study is to present our institutional series of ACDF using the VariLift-C® device, and further delineate the outcomes of cases where multiple levels were fused in one operation or one or more levels were operated in the context of a prior fusion. Our series is the largest to date with these devices, and the first to address their advantage in being used for multiple levels, non-contiguous levels and in prior fused nearby levels.

## Materials and methods

The primary objective of this retrospective chart review was to identify the safety and efficacy of cervical interbody fusion using the expandable VariLift-C® device for consecutive patients undergoing ACDF. All cases were performed by the senior author (J.A.) at a US-based academic institution. We hypothesized that by using the VariLift-C® cervical interbody fusion device for cases requiring multi-level fusion or who had a prior fusion, patients would have less invasive and protracted surgery compared to standard devices, which require the additional use of anterior cervical plating methods. Institutional Review Board approval was obtained prior to study commencement (IRB Protocol #: 2018P000192), with waiver of patient consent due to the retrospective nature of this work.

All patients who underwent cervical fusion with VariLift-C® between 2013 and 2018 were identified. All patients with at least one-month follow-up were eligible. Infectious, traumatic or metastatic causes were excluded from the study. Subgroup analysis was performed for patients undergoing fusion of more than one level and those that had already undergone cervical fusion on the same or a different level in the past.

All surgeries were performed using a standard ACDF procedure. After proper neck preparation and dissection, and identification of the correct cervical spine levels with intraoperative X-rays, the interbody space was decompressed, by excising the disc and endplate tissue. The correct size of VariLift-C® device was selected, and then two devices were inserted bilaterally and expanded in situ using direct and fluoroscopic vision. Finally, the graft material (local autograft mixed with a small amount of demineralized bone matrix allograft) was placed within and over the devices, followed subsequently by surgical closure.

The following variables were collected: Patient’s age at the time of the procedure, gender, preoperative symptoms (neck pain, upper extremity radiculopathy, myelopathy, bowel or bladder dysfunction, and gross neurologic deficit), smoking history, past medical history, and previous surgery of the cervical spine. Regarding the operative details, we collected the segments implanted with VariLift-C® and, for prior-fused cases, the presence of adjacent segment disease (ASD) or failed fusion. Outcomes were recorded based on the clinical condition of the patient on follow-up. An excellent outcome was defined as no residual symptoms on last follow-up. Improvement was defined as substantial decrease in symptom severity as reported by the patient. Symptom worsening or no improvement was also recorded. Complications were recorded, including cases of transient vocal cord paresis, new onset neurologic deficit, or the need for reoperation.

Patient baseline and operative characteristics are reported using descriptive statistics. Continuous variables are reported as means and standard deviations (SD) or medians and interquartile ranges (IQR), depending on data normality. Categorical variables are reported as frequencies and percentages.

## Results

Seventy-one patients with VariLift-C® (Wenzel Spine, Austin, TX) ACDF procedures were identified, including a total of 108 operated cervical levels. Of those, 39 (54.9%) were single-level and 32 (45.0%) were multi-level, while 14 (19.7%) were reoperations. The mean age (± SD) of all patients was 50.3 ± 11.4 years, and the mean (± SD) postoperative follow-up was 6.5 ± 10.7 months (range six weeks to six years).

Baseline patient characteristics are reported in detail in Table 1 for the entire series, as well as the subgroups of multilevel and reoperative procedures respectively.

**Table 1:** Patient demographic characteristics. HTN: Hypertension; N: Number of patients; OSA: Obstructive sleep apnea; PMH: Past medical history; SD: Standard deviation.

Baseline Characteristics	Total Patients, % (N = 71)	Subgroup Analysis	
		Multilevel procedures, % (n = 32)	Revision procedures, % (n = 14)
Age at surgery, years (mean ± SD)	50.3 ± 11.4	52.6 ± 10.1	50.7 ± 9.7
Gender, male	40 (56.3%)	23 (71.9%)	6 (42.9%)
Preoperative symptoms			
Neck pain	45 (63.4%)	18 (56.3%)	9 (64.3%)
Arm radicular symptoms	59 (83.1%)	29 (90.6%)	11 (78.6%)
Leg symptoms	7 (9.9%)	4 (12.5%)	1 (7.1%)
Bowel/bladder dysfunction	2 (2.8%)	2 (6.3%)	1 (7.1%)
Neurologic deficit	30 (42.3%)	12 (37.5%)	2 (14.3%)
Smoking	20 (28.2%)	6 (18.8%)	3 (21.4%)
Past medical history			
Lumbar surgery	4 (5.6%)	1 (3.1%)	1 (7.1%)
Diabetes mellitus	9 (12.7%)	5 (15.6%)	1 (7.1%)
Obesity	2 (2.8%)	1 (3.1%)	0 (0%)
HTN	18 (25.4%)	6 (18.8%)	2 (14.3%)
OSA	6 (8.5%)	0 (0%)	0 (0%)
Dyslipidemia	11 (15.5%)	8 (25%)	3 (21.4%)
Hyper- or hypothyroidism	8 (11.3%)	3 (9.4%)	1 (7.1%)
Depression or anxiety	15 (21.1%)	5 (15.6%)	4 (28.6%)
Osteoarthritis	2 (2.8%)	2 (6.3%)	1 (7.1%)
Non-spinal orthopedic surgery	4 (5.6%)	2 (6.3%)	1 (7.1%)

Operative details are delineated in Table 2.

**Table 2:** Patient operative characteristics. N: Number of patients; SD: Standard deviation.

Fusion Levels	Total Patients, % (N = 71)	Subgroup Analysis	
		Multilevel procedures, % (n = 32)	Revision procedures, % (n = 14)
Spinal segments fused			
C2-C3	1 (1.4%)	1 (3.1%)	1 (7.1%)
C3-C4	5 (7%)	4 (12.5%)	0 (0%)
C4-C5	16 (22.5%)	11 (34.4%)	6 (42.9%)
C5-C6	43 (60.6%)	27 (84.4%)	3 (21.4%)
C6-C7	34 (47.9%)	24 (75%)	4 (28.6%)
C7-T1	9 (12.7%)	2 (6.3%)	5 (35.7%)
Number of segments (mean ± SD)	1.5 ± 0.6	2.2 ± 0.4	1.4 ± 0.6
1-level	39 (54.9%)	NA	10 (71.4%)
2-level	27 (38%)	27 (84.4%)	3 (21.4%)
3-level	5 (7%)	5 (15.6%)	1 (7.1%)
Reoperation	14 (19.7%)	4 (12.5%)	14 (100%)
Single level reoperation	10 (14.1%)	NA	10 (71.4%)
Multilevel reoperation	4 (5.6%)	4 (12.5%)	4 (28.6%)
Reoperation reason			
Fusion extension	11 (78.6%)	4 (100%)	11 (78.6%)
Same level revision	3 (21.4%)	0 (0%)	3 (21.4%)

Overall, the most common levels implanted were C5-C6 and C6-C7, with 60.6% and 47.9% of patients undergoing fusion of that level respectively. Fourteen patients (19.7%) had a previous cervical spinal operation. Of these, 28.6% had a failed fusion, requiring reoperation at the same level, and 71.4% had developed adjacent segment disease, requiring surgery on levels other than those initially fused. Mean (± SD) operative time, from incision to wound closure for first-time procedures was 86.7 ± 20.5 minutes for single-level, 121.2 ± 20.9 minutes for 2-level, and 138.0 ± 12.3 minutes for 3-level fusions. In the reoperative group, operative time was 97.6 ± 32.6 minutes for fusion extension on the adjacent segment, and 101.7 ± 16.8 minutes for same-segment revisions.

**Figure 1:**
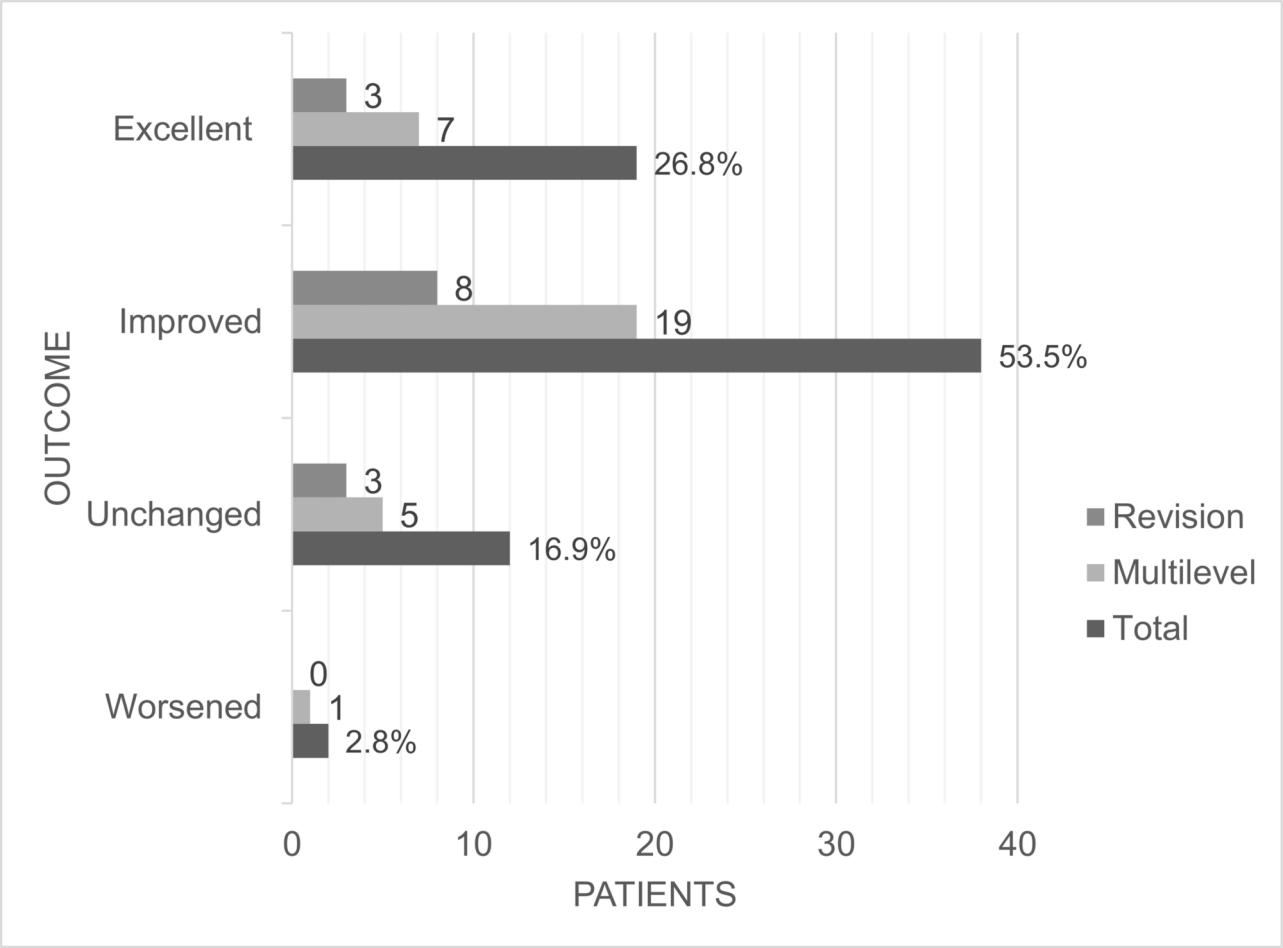
Postoperative outcomes after cervical VariLift-C® implantation, after variable follow-up period, subdivided into total patient cohort, the multilevel subgroup and the reoperative subgroup. Outcomes are compared to the preoperative patient condition. An excellent outcome indicates no residual symptoms.

Figure 1 depicts patients outcomes, assessed on their last follow-up for the entire series and its subgroups.

**Figure 2:**
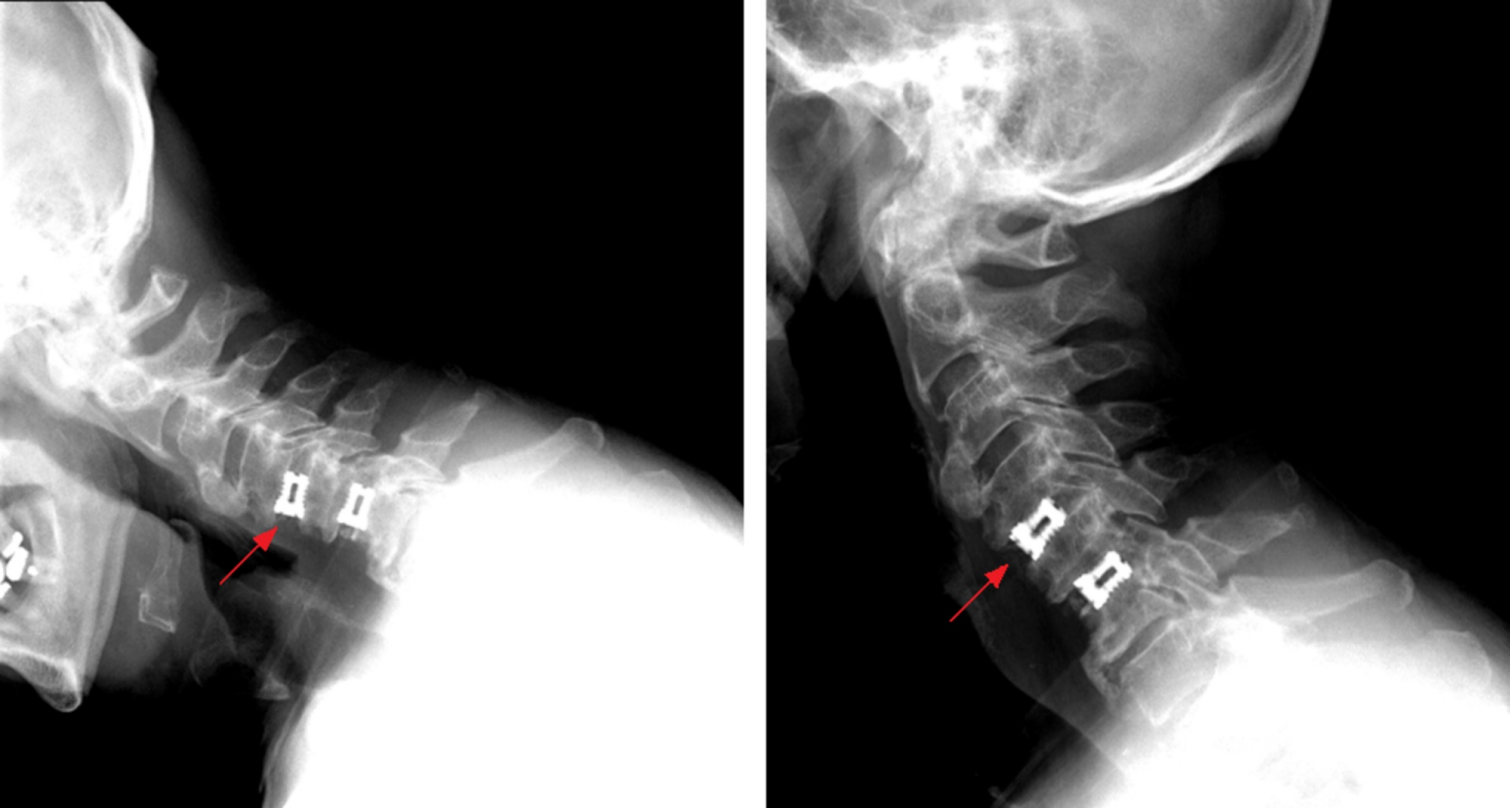
Dynamic lateral cervical X-ray imaging on six-week follow-up after VariLift-C® implantation. The wedge-shape of the implant (arrows) is identifiable and contributes to the correction of sagittal alignment. (Left) Flexion, (Right) Extension.

A total of 80.3% of the total cohort had significant improvement in their symptoms compared to their preoperative state, with 26.8% having an excellent outcome with no residual symptoms. In the multiple-level subgroup, a total of 81.3% reported improvements, with 21.9% having no residuals, and for the reoperative group, 78.5% improved, with 21.4% having no residual symptoms. The mean follow-up duration was 6.5 months (range six weeks to six years). A sample case is presented in Figure 2.

Table 3 provides a breakdown of complications encountered among the different groups of patients undergoing VariLift operations.

**Table 3:** Complications after VariLift cervical procedures among the single- and multi-level and index surgery and reoperation categories. n: Number of patients; CSF: Cerebrospinal fluid; OR: Operative room.

Complications	Subgroup Analysis		
	Single level procedures, % (n = 39)	Multiple level procedures, % (n = 32)	Revision procedures, % (n = 14)
Index surgery	n = 29	n = 28	na
CSF Leak	0 (0)	0 (0)	na
Dysphagia	1 (3.4)	0 (0)	na
Dysphonia	1 (3.4)	1 (3.6)	na
Wound Infection	1 (3.4)	0 (0)	na
Hematoma	1 (3.4)	0 (0)	na
Neurologic deterioration	4 (13.8)	4 (14.3)	na
Esophageal injury	0 (0)	0 (0)	na
Return to OR	6 (20.7)	1 (3.6)	na
30-day readmission	2 (6.9)	0 (0)	na
Reoperation	n = 10	n = 4	n = 14
CSF Leak	0 (0)	0 (0)	0 (0)
Dysphagia	0 (0)	0 (0)	0 (0)
Dysphonia	0 (0)	0 (0)	0 (0)
Wound Infection	0 (0)	0 (0)	0 (0)
Hematoma	0 (0)	0 (0)	0 (0)
Neurologic deterioration	2 (20)	0 (0)	2 (14.3)
Esophageal injury	0 (0)	0 (0)	0 (0)
Return to OR	2 (20)	0 (0)	2 (14.3)
30-day readmission	0 (0)	0 (0)	0 (0)

Six patients that had undergone a single-level index procedure, had to return to the operating room. Two returned acutely in the 30-day postoperative period, due to a retropharyngeal hematoma and a case of severe swallowing difficulty, respectively. Two other cases had adjacent segment disease requiring reoperation 12 and 50 months postoperatively, respectively. Finally, two patients had same-level procedures after three months and 21 months, due to neurologic worsening and spinal cord compression. No pseudoarthrosis or hardware failure was noted in any of these cases. One patient that had undergone a multilevel index VariLift procedure had to be taken back to the operating room on postoperative day 1 for revision of the instrumentation, due to acutely worsening neurologic function. Additionally, two reoperative, single-level Varilift patients proceeded to worsen neurologically and eventually received spinal cord stimulators, four and nine months after the Varilift operation, respectively.

## Discussion

This retrospective case series reports on a single center experience with efficacy and safety of a standalone, expandable cervical interbody fusion device, focusing on multiple-level fusions and in reoperations for patients who had prior ACDF. These patients present more challenging surgical environments and our experience using VariLift-C® allowed us to avoid the removal of existing anterior plates or navigate screw placement with pre-existing anchored cages. We believe the device characteristics helped us achieve excellent technical and clinical performance in our patients, corroborating and expanding on previous literature on expandable devices for ACDF [2-4].

Prior to implantation, the VariLift-C device is cylindrical with self-tapping threads, which allows for smooth, direct introduction of both devices next to each other within the intervertebral space. After correct positioning the device is expanded with a small internal disk. This expansion transforms the geometry from cylindrical to a lordotic wedge (Figure 2) and enables further purchase on the vertebral body endplates. We have found the bone graft chamber to be advantageous in that fenestrations open on all sides allow graft contact with the endplates.

The use of this implant in our patients has given us controlled insertion, immediate immobilization and restoration of the disk height, and normal spinal alignment (Figure 2). Expandable cages have previously been shown to be biomechanically [5] and clinically equivalent to nonexpendable cages with bone autografting for various indications in the cervical spine, including degenerative [2], tumor [6], and infectious disorders [7]. Auguste et al. showed expandable devices in the cervical spine also provide adequate structural support outcomes, with low rates of complication [2]. To our knowledge, this is the first clinical report of VariLift-C; greater evidence has been reported for VariLift in the lumbar spine demonstrating excellent outcomes in lumbar curvature correction [8], as have other standalone devices [9-12].

In this study, we noted substantial improvement in patient symptoms, with about 80% of patients reporting improvement, and 26% being completely symptom-free on follow-up. Importantly, multiple-segment and reoperative procedures showed similarly favorable outcomes, despite their propensity for less ideal results historically. No patient undergoing a multilevel procedure required reoperation during follow-up, while two reoperative cases progressed further, requiring same-segment surgery in one case and spinal cord stimulator insertion in two, to address symptoms. These results corroborate the findings by previous authors about the efficacy of expandable devices to provide improvement in both pain and neurologic symptoms [2-4,13], and expand on the literature regarding standalone cages. The results also compare favorably with typical bone and plate ACDF. In the present study, 12.8% of the total patients experienced neurologic deterioration postoperatively. Vocal cord paresis was a significant source of postoperative issues, accounting for three postoperative complications in the series, and presenting with hypophonia, swallowing difficulty or breathing problems. It required further management in one case and was transient in the rest. Depending on the study referenced, incidence of transient post-operative dysphagia for ACDF with an anterior plate can range from 33.3% to 46% [14,15] and persistent dysphagia ranging from 2.5% to 12.1% [16,17]. In contrast, our experience was a 1.3% overall rate of dysphagia. In index ACDF, transient dysphonia (recurrent laryngeal nerve palsy) can be as high as 2.9% [18], and in revision cases this jumps to 14.1% with a 95% confidence interval of 10% to 19% [19]. Our series had a 2.6% rate of transient dysphonia, with 2.8% in single-segment index procedures, and 3.6% in multilevel index procedures, while revision procedures had no such complications.

While ACDF is considered safe and effective in managing single-level cervical degenerative disease [20], there is controversy regarding multiple-level procedures [21,22]. Traditional techniques create the need for a longer, more longitudinal surgical exposure and significant tissue manipulation to accommodate multiple-segment anterior plating. Additionally, anterior plating is added to conventional bone graft to facilitate fusion and maintain normal cervical curvature, but is associated with postoperative complications, such as vocal cord paresis and dysphagia [23,24]. Standalone expandable devices like the VariLift-C® obviate the need for anterior plating, which could pose a substantial advantage. However, VariLift-C® has certain limitations. It is not appropriate for standalone use in the presence of instability. Additionally, standalone devices have previously been shown to have increased risk for subsidence and regional deformity than traditional techniques [25]. However, a registered prospective clinical trial has identified no cases of subsidence, 12 months after VariLift-C implantation in 26 patients [26]. Therefore, proper patient selection to account for all the above factors, combined with proper device placement to minimize risk for complications is imperative to obtain the best results with this device.

We recognize that our study has certain limitations. As with all retrospective case series, it has the inherent limitation of potential selection bias. Additionally, no anterior plating control group was included for comparison. Long-term follow-up was limited, with many patients only having one month to six weeks of follow-up, because they were not required to return for follow-up unless they experienced symptomatology requiring evaluation. Therefore, full radiographic fusion rates were not available because long-term follow-up images would only be available in patients more likely to have symptom recurrence that required imaging. Furthermore, because of the lack of a pain or symptom severity scale, outcomes are reported based on patient descriptions of their symptoms in follow-up notes and the lack of further documented operative procedures.

## Conclusions

In conclusion, this retrospective case series found the standalone, expandable cervical interbody fusion device VariLift-C® to have excellent technical performance characteristics and to be effective in providing favorable outcomes, with high rates of symptomatic improvement and few complications, particularly in patients requiring extension from prior ACDF or multiple, non-contiguous segment fusions. Our study showed both the efficacy and safety of VariLift-C®, while indicating advantages over anterior plating techniques for such patients. Future studies should prospectively utilize standardized scales such as the visual analog pain scale and the Oswestry Disability Index, as well as fusion and subsidence evaluations, to compare with other techniques more formally.
